# Early Onset Cerebral Infarction in Schimke Immuno-Osseous Dysplasia

**Published:** 2018

**Authors:** Amir HOSSEIN BABAEI, Soroor INALOO, Mitra BASIRATNIA, Ali DERAKHSHAN

**Affiliations:** 1 Medical Student, Student Research Committee, Shiraz University of Medical Sciences, Shiraz, Iran; 2 Department of Pediatric Neurology, Neonatal Research Center, Shiraz University of Medical Sciences, Shiraz, Iran; 3 Department of Pediatrics Nephrology, Shiraz Nephrology-Urology Research Center, Shiraz University of Medical Sciences, Shiraz, Iran

**Keywords:** Schimke immuno-osseous dysplasia, Cerebrovascular disorders, Renal insufficiency, Nephrotic syndrome

## Abstract

Schimke Immuno-osseous Dysplasia (SIOD) is a rare autosomal recessive disease caused by a biallelic mutation in SMARCAL1 gene. Typical findings in SIOD include spondylo-epiphyseal dysplasia, steroid resistance nephrotic syndrome, progressive renal failure, T-cell immunodeficiency, bone marrow failure, and cerebral infarction. In this case report, we describe a 9-yr-old girl who presented with failure to thrive in infancy. Nephrotic syndrome was diagnosed at the age of four years. She had three episodes of admission with cerebral stroke due to moyamoya syndrome. In the last admission at Namazi Hospital, Shiraz, southern Iran, in October 2016, she had new cerebral ischemia, developed seizure, and finally died.

## Introduction

Schimke Immuno-Osseous Dysplasia (SIOD) (OMIM: 242900) is a rare autosomal recessive disease caused by a biallelic mutation in *SMARCAL1* gene (Gene ID: 50485; NG_009771.1; OMIM: 606622). The incidence of SIOD is not clear, but it has been estimated to be 1 in every 1000000 to 3000000 live births in the United States (1).

Common findings in SIOD include spondylo-epiphyseal dysplasia (SED), Steroid Resistance Nephrotic Syndrome (SRNS), progressive renal failure, and T-cell immunodeficiency. Atherosclerosis, cerebral infarction, Transient Neurologic Attacks (TNAs), hypothyroidism, and corneal opacity are also associated with SIOD. Bone marrow failure, autoimmune disease, non-Hodgkin lymphoma, and osteosarcoma has also been reported in these patients previously (1-3).

Up to now, only two patients with SIOD have been reported in Iran (2, 3).

Herein, we describe a 9-yr-old girl with SIOD who presented initially with failure to thrive in infancy. Nephrotic syndrome was diagnosed when she was 4 yr old. 

## Case report

In October 2016, a 9-yr-old girl with chief complaint of nausea, vomiting, lethargy, and decreased level of consciousness referred to the Neurology Department of Namazi Hospital, Shiraz, southern Iran.

The proband was the third offspring of healthy consanguineous parents (cousins). She had a healthy brother and sister. She was delivered through cesarean section due to oligohydramnios (birth weight = 2700 gr, body length = 48 cm). She had a short neck and trunk, pectus carinatum, and kyphosis. The first problem of the patient occurred at five months of age when growth retardation was detected in routine workup. Dental age was also delayed compared to chronological age. Indeed, bone survey showed delayed bone age, J-shaped sella, periarticular and diffused osteopenia, and flattening of thoracic vertebrae. She developed urinary tract infection when she was 11 months old. In voiding cystourethrogram, bilateral vesicoureteral reflux was diagnosed. Kidneys, ureters, and urinary bladder ultrasonography and renal scintigraphy were normal.

At the age of 4 yr, urine analysis showed proteinuria for the first time and after more workups, nephrotic syndrome was confirmed.

She had no new problems up to the age of 6 yr when she developed sudden onset right upper extremity paresthesia and weakness. Brain MRI was performed and showed ischemic and hemorrhagic infarct in the left parieto-occipital and left caudate lobe. Brain Magnetic Resonance Angiography (MRA) also revealed significant wall irregularity of both internal carotid arteries, left Middle Cerebral Artery (MCA), basilar artery, and left Posterior Cerebral Artery (PCA). MRI of the cervical spine was normal.

**Figure 1:**
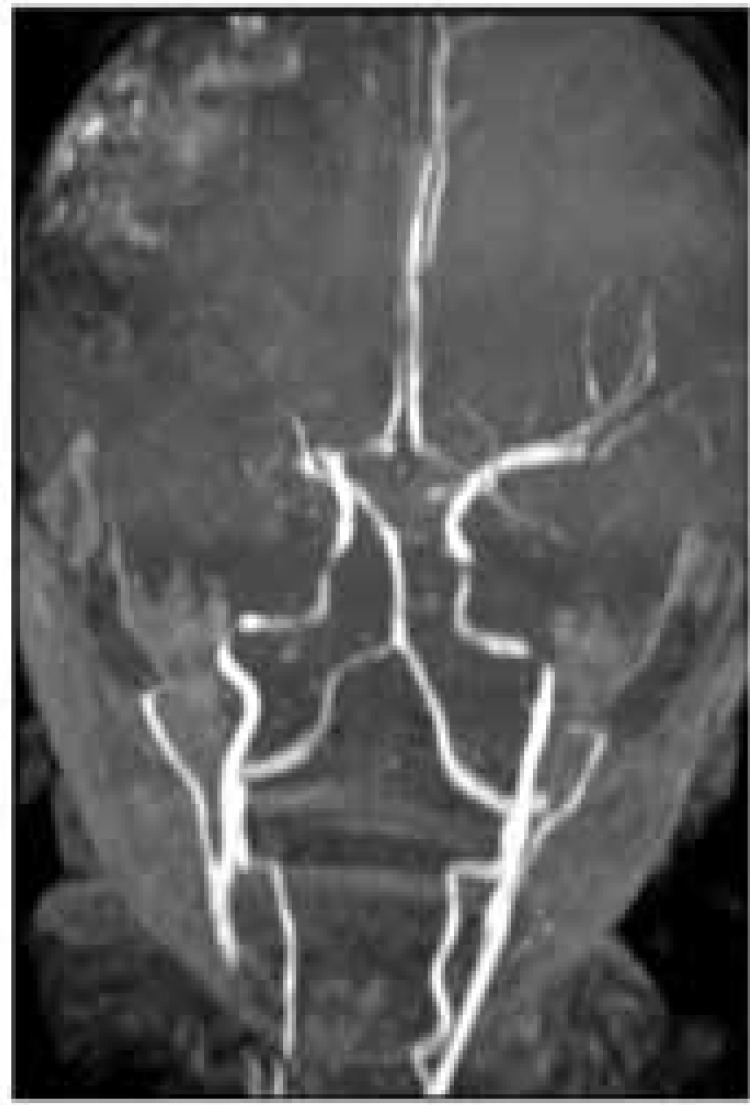
Patient’s brain Magnetic Resonance Angiography (MRA) on the second admission

Two years later, she developed slurred speech and paresthesia, and weakness was progressed to her lower extremities. Brain MRI was performed again revealing new acute ischemic infarction in the right temporoparietal lobe. Encephalomalacia with surrounding gliosis was also noted involving the left parieto-occipital lobe because of the old infarction. Complete obstruction of the right MCA from the proximal part was seen in brain MRA. There was also evidence of significant wall irregularity and stenosis in basilar artery, PCA, Anterior Cerebral Artery (ACA), internal carotid arteries, and vertebral arteries. These findings were in favour of moyamoya syndrome (Figure 1). Brain Magnetic Resonance Venography (MRV) was normal. (Figure1).

**Figure 2:**
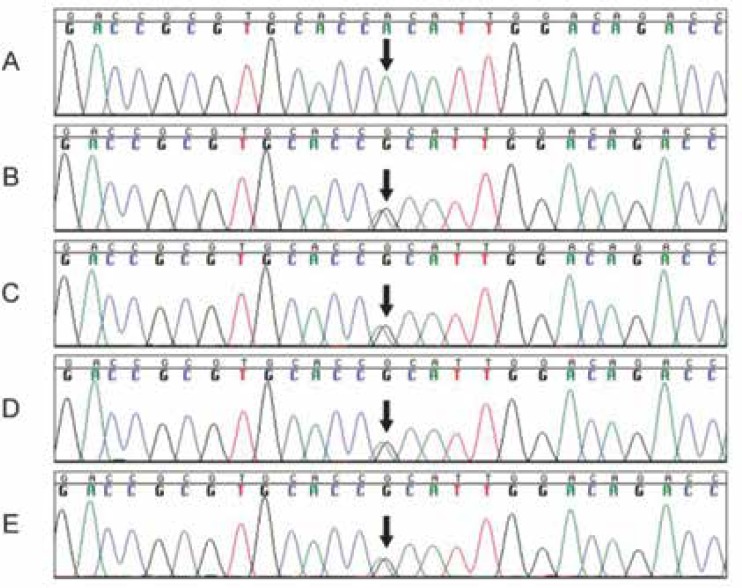
The chromatogram indicates the homozygous mutation of SMARCAL1 gene in the proband. Panel (A), from the proband illustrating AA sequence (c.2459) leading to the substitution of Arginine (CGC) at position 820 in the protein by histidine (CAC). Panels (B, C, D, and E) show heterozygote (G/A) pattern of the same section from the patient’s parents and siblings

Analysis of *SMARCAL1* gene was performed for detection of mutations (1), which revealed a homozygous nonsynonymous homozygous mutation c. (2459G>A). This mutation was found by direct sequencing of both sense and antisense strands of the 16 coding and 2 noncoding exons of *SMARCAL1* (Figure 2).

**Figure 3:**
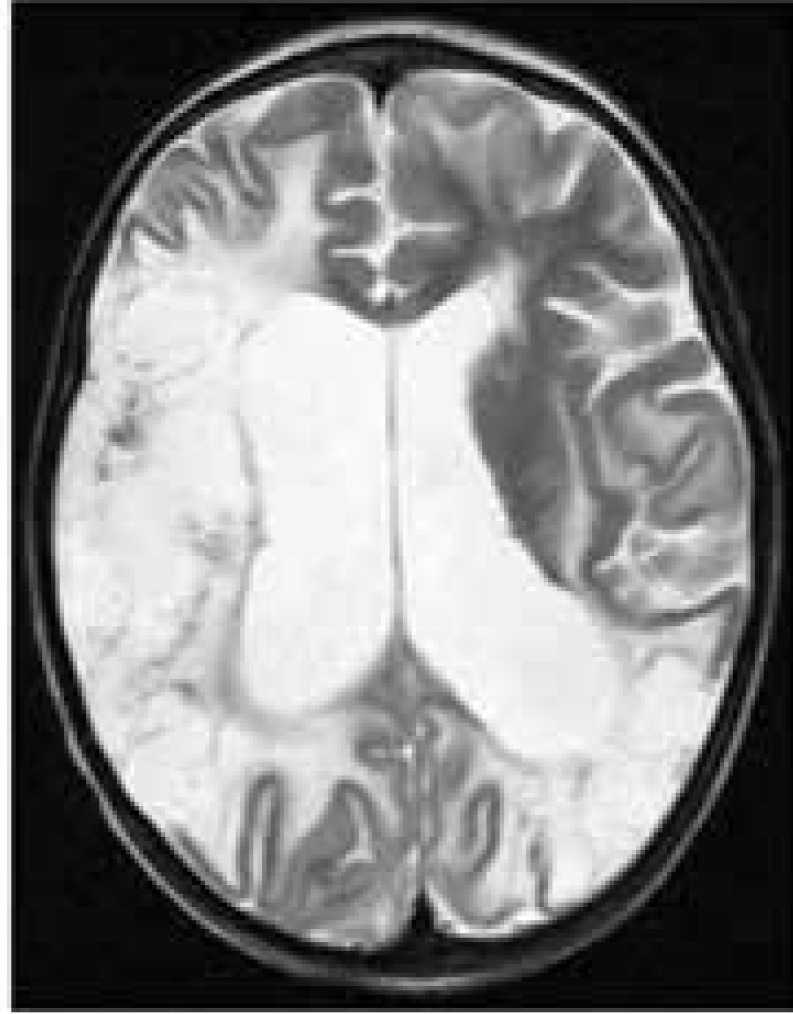
Patient’s brain MRI on the last admission

Her last admission was at the age of 9 yr due to nausea, vomiting, lethargy, and decreased level of consciousness (Glasgow Coma Scale 7/15) at the Namazi Hospital, Shiraz, southern Iran, in October 2016. In the emergency room, she had four episodes of Generalized Tonic-Clonic (GTC) seizure, controlled by phenytoin and diazepam. The Electroencephalogram (EEG) was in favor of diffused brain suppression. Indeed, brain MRI noted encephalomalacia in the territory of right and left MCA with involvement of the whole right parietal lobe due to previous infractions. Besides, laminar necrosis was detected in the right parietal lobe. There was also evidence of a hematoma in the right parietal lobe. Ventricular dilatation was observed due to brain parenchymal atrophy and volume loss (Figure 3). She also had hyperkalemia, metabolic acidosis and anuria, and high creatinine level due to End-Stage Renal Disease (ESRD). Hemodialysis was not done owing to her parents’ dissatisfaction with insertion of central veins catheter, and she just received conservative therapy. Finally, she expired because of cardiopulmonary arrest following pulmonary hemorrhage. The laboratory findings of the patient have been presented in Table 1.

Informed consent form was obtained from parents before participation in the study in accordance. Ethics Committee of Shiraz University of Medical Sciences approved the study.

## Discussion

We presented a 9-yr-old girl with failure to thrive and SRNS who developed cerebral infarction at the age of 6 yr as a complication of SIOD.

The exact etiology of SIOD has not been clearly recognized. Although about 55 different mutations in *SMARCAL1* gene have been identified in association with SIOD; only 50%-60% of individuals with SIOD have detectable mutations in this gene (1). Mutations in other unidentified genes can also cause SIOD. The SMARCAL1 gene is located on chromosome 2q35 and encodes the HepArelated protein (HARP), a member of the Sucrose Non-Fermentable (SNF2) family of ATPase that acts as a chromatin remodeler within multi-protein complexes. This protein is an ATP-driven annealing helicase involved in a wide range of biological functions; including transcription, DNA replication, and DNA repair (1).

The mean lifespan of individuals with SIOD is 11 yr. The most important causes of death include infection (23%), stroke (13%), pulmonary hypertension and congestive heart failure (13%), renal failure (11%), complications of organ transplantation (9%), lymphoproliferative disease (4%), gastrointestinal complications (4%), respiratory failure (4%), bone marrow failure (2%), non-Hodgkin lymphoma (2%), pancreatitis (2%), and other unreported cases (13%) (4).

The first laboratory abnormality in our patient was proteinuria in nephrotic range and SRNS; however, kidney biopsy was not done due to her parents’ dissatisfaction. Angiotensin-Converting Enzyme Inhibitors (ACE-I) and prednisolone were prescribed for the patient, but the patient didn’t go to remission

Nowadays, SIOD children’s life expectancy has increased due to recent advances in transplantation and dialysis. Yet, cerebrovascular disease has become an important cause of morbidity and mortality.

*SMARCAL1* gene mutation can result in a broad range of neurological manifestations in SIOD ranging from severe migraine-like headaches and TNAs to ischemic events. TNAs are frequently focal and do not have an ischemic origin (4). On the other hand, cerebral ischemic events are precipitated by hypertension occurring due to administration of high doses of steroids or even disease progression. Patients with TNAs or strokes usually have diffuse progressive cerebral arteriosclerosis, while those with isolated migraine-like headaches do not.

“Cerebrovascular pathology in SIOD could result from several factors. Atherosclerosis could result from the combination of hypertension and hyperlipidemia secondary to renal disease with immune dysfunction” (5). “Additionally, vascular changes include focal intimal lipid deposition, focal myointimal proliferation, macrophage invasion, foam cells, fibrous transformation, and calcium deposits” (6). Another possible mechanism of microscopic vascular pathology is decreased elastin expression, which leads to disorganized internal elastic lamina and medial and intimal hyperplasia (7).

Seizure is another neurological manifestation of SIOD. These patients had EEG abnormalities, such as excessive background slowing and foci of rhythmic and arrhythmic slowing. Nonetheless, a small number of them experienced clinical seizure before occurrence of cerebral ischemia. The particular pathophysiology of seizure in these patients is not clear, but cortical microgenesis has been associated with primary generalized epilepsy and partial epilepsy (8). One of the suspicious causes of early-onset seizure after acute ischemia with cortical and subcortical involvement is increased extracellular concentrations of glutamate that leads to recurrent epileptiform-type neuronal discharges. Late-onset seizures can occur owing to gliosis and development of a meningocerebral cicatrix. Hyperexcitability of neurons subsequent to changes in membrane properties, selective neuronal loss, deafferentation, and collateral sprouting may be other causes of seizures (9).

**In conclusion,** SIOD should be considered in children with growth retardation, SRNS, skeletal abnormalities, and neurological symptoms. Patients suspected to have SIOD, even before development of the full picture, should be carefully monitored for neurological symptoms, such as migraine-like headaches, mood and memory problems, seizure, and cerebrovascular accident. Moreover, evaluation of cerebral vasculature and preventive vascular intervention in patients with cerebrovascular abnormality may be helpful in preventing early-onset cerebral infarction.

**Table 1:** The laboratory data of the patient on admission

**Laboratory tests**	**Measured level**	**Normal values**
Hemoglobin (gr/dl)	8.5	12-14.5
White blood cells (10^3^ /μl)	6300	3400-10800
Polymorph (10^3^ /μl)	5008 (79.5%)	1500-8500
Lymphocyte (10^3^ /μl)	1071 (17%)	1500-6500
Monocyte (10^3^ /μl)	220 (3.5%)	0-800
Platelet (10^3^ /μl)	95000	150000-450000
BUN (mg/dl)	40	6-21
Creatinine (mg/dl)	6	0.6-1.2
Ca (mg/dl)	8.9	8.8-10.2
P (mg/dl)	7.1	2.7-4.5
ALT (U/L)	19	7-45
AST (U/L)	35	8-50
Alk Phosphatase (U/L)	465	183-402
Total Bilirubin	0.3	0.2-1.2
Direct Bilirubin	0.1	Under 0.4
Na (meq/l)	149	136-145
K (meq/l)	6.1	3.5-5.5
TG (mg/dl)	149	Under 150
Cholesterol (mg/dl)	153	Under 200
Albumin (mg/dl)	1.6	3.5-5.2
Total protein (mg/dl)	4.6	6.6-8.8
TSH (mIu/ml)	3.24	0.35-4.94
T4 (ng/dl)	5.68	4.78-11.72
T3 (ng/dl)	0.75	0.58-1.59
24-hour urine protein (mg)	1100	Under 140
T cell (CD+3) (%)	32	55-78
T cell (CD+4) (%)	6	27-53
T cell (CD+8) (%)	21	19-34
T cell (CD+16) (%)	10	9.2-19.7
T cell (CD+19) (%)	58	10-31
(CD4/ CD8)	0.29	0.9-2.6
ESR (mm/hr)	95	3-13
CRP	13	Under 6
Urine analysis	pH: 1.013 pro: 4+ blood: 3+ glucose: 3+ ketone: 1+ RBC: 4-6 WBC: 3-4 Bacteria: moderate Nitrite: negative Leukocyte esterase: negative	

BUN, blood urea nitrogen; Ca, calcium; AST, aspartate transaminase; ALT, alanine transaminase; Alk phosphatase, alkaline phosphatase; Na, sodium; K, potassium; TG, triglyceride; TSH, thyroid stimulating hormone; T4, thyroxine; T3, triiodothyronine; ESR, erythrocyte sedimentation rate; CRP, C-reactive protein.

## References

[1] Santangelo L, Gigante M, Netti GS, Diella S, Puteo F, Carbone V (2014). A novel SMARCAL1 mutation associated with a mild phenotype of Schimke immuno-osseous dysplasia (SIOD). BMC Nephrol.

[2] Basiratnia M, Baradaran-Heravi A, Yavarian M, Geramizadeh B, Karimi M (2011). Non-hodgkin lymphoma in a child with schimke immuno-osseous dysplasia. Iran J Med Sci.

[3] Basiratnia M, Fallahzadeh MH (2007). Schimke immuno-osseous dysplasia. Saudi Med J.

[4] Morimoto M, R.A. Pagon (1993-2016). Schimke Immunoosseous Dysplasia. GeneReviews(R).

[5] Zieg J, Krepelova A, Baradaran-Heravi A, Levtchenko E, Guillén-Navarro E, Balascakova M (2011). Rituximab resistant evans syndrome and autoimmunity in Schimke immuno-osseous dysplasia. Pediatr Rheumatol Online J.

[6] Clewing JM, Antalfy BC, Lücke T, Najafian B, Marwedel KM, Hori A (2007). Schimke immuno-osseous dysplasia: a clinicopathological correlation. J Med Genet.

[7] Morimoto M, Yu Z, Stenzel P, Clewing JM, Najafian B, Mayfield C (2012). Reduced elastogenesis: a clue to the arteriosclerosis and emphysematous changes in Schimke immuno-osseous dysplasia?. Orphanet J Rare Dis.

[8] Deguchi K, Clewing JM, Elizondo LI, Hirano R, Huang C, Choi K (2008). Neurologic phenotype of Schimke immuno-osseous dysplasia and neurodevelopmental expression of SMARCAL1. J Neuropathol Exp Neurol.

[9] Camilo O, Goldstein LB (2004). Seizures and epilepsy after ischemic stroke. Stroke.

